# A review on *Trichomonas vaginalis* infections in women from Africa

**DOI:** 10.4102/sajid.v36i1.254

**Published:** 2021-06-10

**Authors:** Nonkululeko Mabaso, Nathlee S. Abbai

**Affiliations:** 1 School of Clinical Medicine, College of Health Sciences, University of KwaZulu-Natal, Durban, South Africa

**Keywords:** *Trichomonas vaginalis*, Africa, epidemiology, drug resistance, health complications

## Abstract

**Background:**

Trichomoniasis is the most common sexually transmitted infection (STI) with an estimated annual incidence of 276.4 million cases globally and about 30 million cases in sub-Saharan Africa. Trichomoniasis has been found to be associated with various health complications including pelvic inflammatory disease (PID), significant pregnancy complications, cervical cancer, prostatitis, infertility and the acquisition of human immunodeficiency virus (HIV).

**Aim:**

Despite being a highly prevalent infection in the African continent, there is no review article published that solely focusses on *Trichomonas vaginalis* (*T. vaginalis*) infections in women from Africa. This review aims to fill this gap in the literature.

**Method:**

An electronic search of online databases was used to identify and extract relevant research articles related to the epidemiology, health complications and treatment associated with *T. vaginalis* in women from Africa.

**Results:**

Within the African continent, South Africa has reported the highest prevalence rate for this infection. A combination of sociodemographic, behavioural and biological factors has been shown to be associated with infection. *Trichomonas vaginalis* infection is associated with the acquisition of HIV, cervical cancer and PIDs in various female populations across the continent. Emerging patterns of resistance to metronidazole have been reported in women from South Africa. Currently, there is no effective vaccine against this pathogen despite efforts at vaccine development.

**Conclusion:**

Based on the high prevalence and health consequences associated with *T. vaginalis*, there is a need for improved screening programmes that will lead to early diagnosis, detection of asymptomatic infections and effective treatment regimens.

## Introduction

*Trichomonas vaginalis* (*T. vaginalis*) is an anaerobic parasitic protozoan that causes the sexually transmitted infection (STI) trichomoniasis.^1,2^ In sub-Saharan Africa, about 30 million infections occur annually.^3^ The estimated prevalence in South African women aged 15–24 years ranges from 3.1% to 20%.^4^ Moodley et al. (2015), De Waaij et al. (2017) and Morikawa et al. (2018) reported prevalence rates of 15.3%, 20.0% and 20.2%, respectively, amongst South African women.^5,6,7^ *Trichomonas vaginalis* infection prevalence in pregnant women ranges from 17% to 20% in Africa, 16% to 53% in the United States and 0.8% in Asia.^8^ Studies that have been conducted in South African pregnant women have reported prevalence rates of 15.3%^5^ and 20.2%.^7^ Several risk factors associated with *T. vaginalis* infection have been reported including older age, co-infection with other STIs,^9,10^ sexual behaviour, socioeconomic status and phase of menstrual cycle.^9^ In women from Africa, older age, marital status, multiple sex partners, poor hygiene and socioeconomic status have been shown to be risk factors associated with *T. vaginalis* infection.^1^ Infection with *T. vaginalis* is asymptomatic in about 50% of women.^11^ However, when symptoms arise, women experience vaginal discharge, pruritus and/or dysuria, vaginitis and cervicitis.^3,12^

Trichomoniasis has been found to be associated with various health complications including pelvic inflammatory disease (PID), significant pregnancy complications, cervical cancer, prostatitis and infertility.^13,14^ Significant pregnancy complications include pre-term labour, low birth weight and premature rupture of membranes.^15,16^ *Trichomonas vaginalis* infection has also been associated with a high risk of acquisition and transmission of human immunodeficiency virus (HIV).^3,6^ Reports on metronidazole resistance of *T. vaginalis* strains have been increasing.^1^

Even though metronidazole cure rates are high, clinical treatment failure is challenging.^17^ Most *T. vaginalis* isolates are highly susceptible to metronidazole; however, laboratory resistance and treatment failure have been reported.^18,19^ Reported metronidazole resistance in clinical *T. vaginalis* isolate ranges from 2% to approximately 13%.^20,21,22,23^ In South Africa, metronidazole resistance was reported in 6% (2/30) of the *T. vaginalis* strains isolated from women attending an antiretroviral clinic at the Tshwane District Hospital, Pretoria.^24^ Here, we summarise the epidemiology of *T. vaginalis* across female populations in Africa and discuss health implications and treatment challenges associated with the infection.

## Review methodology

An electronic search of the following databases was conducted: PubMed/MEDLINE and Google Scholar. The search terms included *T. vaginalis*, epidemiology, risk factors, women, treatment and Africa. Boolean terms (AND, OR) were used to separate the keywords, and Medical Subject Headings (MeSH) terms were included during the search. The search was limited to articles published in English. Relevant studies were identified by searching literature from January 1980 to date. The search yielded 14 400 results. Articles were also searched through the ‘Cited by’ search as well as citations included in the reference lists of included articles. Eligibility criteria for selection of articles included being available in full text and related to *T. vaginalis* infection in women from Africa.

## Epidemiology

### Prevalence estimates

#### Global level

Trichomoniasis is the most common STI with an estimated annual incidence of 276.4 million cases globally.

Trichomoniasis prevalence varies greatly in different World Health Organization (WHO) regions.^25^ The WHO stated in 2016 that the highest prevalence estimates for trichomoniasis in women were reported for the African region (11.7%), followed by the region of the Americas (7.7%), Western Pacific (5.6%), East Mediterranean region (4.7%), South-East Asia region (2.5%) and lastly the European region (1.6%).^25^

In 2016, the WHO’s global estimated prevalence for trichomoniasis was 5.3% in women and 0.6% in men.^25^ The lower prevalence of the infection in males is possibly because of the presence of zinc, which is found in the prostatic fluid. Zinc is known to have anti-trichomonal activity *in vitro*, eventually resulting in the inhibition of *T. vaginalis in vivo.*^26^

#### In Africa

Several studies have reported prevalence data on *T. vaginalis* from populations across Africa. A study conducted by Maina et al. (2016) amongst women attending a family planning clinic in Kenya reported a 0.4% prevalence rate for *T. vaginalis.*^27^ In a population of non-pregnant women from Swaziland, *T. vaginalis* was found to be the most prevalent STI (8.4%) in the study population.^28^ The prevalence of *T. vaginalis* in antenatal women from Kenya was reported to be 6.6%.^29^ A more recent study conducted in antenatal women from Tanzania reported a prevalence rate of 7.1% for *T. vaginalis.*^30^ A similar prevalence rate for *T. vaginalis* (7.8%) was reported for antenatal women from Sudan.^31^ Within the African continent, the highest rates of infection have been reported in South Africa. Several studies have reported prevalence data on *T. vaginalis* from South African populations. These studies are described below. Data obtained from a study conducted by Moodley et al. (2002) on women attending a reproductive health clinic in KwaZulu-Natal reported a prevalence rate of 29% for *T. vaginalis.*^32^ In another study conducted on female sex workers from Durban, the prevalence rate for *T. vaginalis* was 20.3%.^33^ Two recent studies reported prevalence rates of 10% and 13%, respectively, for *T. vaginalis* in antenatal women from Durban.^34,35^

According to modelling analysis, sub-Saharan Africa has been identified as the region with a high incidence and prevalence of *T. vaginalis*. Despite data on the prevalence of STIs in countries in sub-Saharan Africa being available, several countries report on the prevalence of syndromes rather than on individual pathogens.^36^ To this end, the true burden of infections in sub-Saharan Africa is not well documented.^36,37^ In addition, the true burden of this infection is not really known as many studies have reported that STIs including *T. vaginalis* are largely asymptomatic.^38,39,40,41^ To obtain accurate prevalence estimates for infection (asymptomatic and symptomatic), diagnostic testing will need to be implemented. However, these tests are not without limitations, as they may be costly for resource-strained settings.^7^

### Risk factors for infection

Several studies have identified risk factors for *T. vaginalis* infection in women. Risk factors for adolescent girls and women attending STI clinics include having sex without a condom, ethnicity (more prevalent within the black race group), multiple sexual partners, greater years of sexual activity, history of STIs and previous infection with *T. vaginalis.*^42,43^

Studies conducted by Abbai et al. (2013) and Naidoo et al. (2014) in women from KwaZulu-Natal, South Africa, found that women at the age of 25 years and younger were at a higher risk of acquiring STIs.^44,45^ Reasons related to younger women being more at risk for infection included behavioural and biological factors.^44^ Abbai et al. (2013) also found that women who were previously diagnosed with an STI have a much greater risk for future infection. Other risk factors for acquiring infection included having a lower level of education.^44,46^ A study conducted by Eshete et al. (2013) in pregnant women from Ethiopia showed that educational status did play a critical role in respect to the increased number of *T. vaginalis* infections.^47^ Mabaso et al. (2020) found that abnormal vaginal discharge was associated with infection. However, it is important to note that abnormal vaginal discharge would not be considered a risk factor for *T. vaginalis* infection; rather it is a symptom of infection and is therefore associated with infection.^35^ Women with *T. vaginalis* infections should be counselled on the use of condoms and the risk of new infections as a result of behavioural practices.

### Diagnosis

#### Microscopic techniques

Wet mount microscopy is the first method to be used for the diagnosis of trichomoniasis, and it remains the most frequently used method.^48,49^ Wet mount examination requires a minimal protozoal concentration of 10^4^ organisms/mL.^49,50^ The sensitivity of wet mount microscopy is between 35% and 80%,^17,48,49,50^ and it is influenced by the experience of the observer, the presence and the concentration of viable and motile trichomonads in the specimen and the period of transportation of the specimen to the laboratory.^49,50^ Wet mount is the simplest and an inexpensive diagnostic test.^10,17,48,50^ However, it is associated with under-diagnosis of the disease because of the low sensitivity^10,50^ and the specimens should be examined immediately after collection because *T. vaginalis* is unstable in conditions outside the body.^13,48^ *Trichomonas vaginalis* retracts its flagella, changes shape (becomes rounder) and loses motility. Therefore, it becomes difficult to distinguish between trichomonads and cells with similar morphology such as white blood cells when there is a delay in specimen examination.^48^

Staining techniques such as acridine orange (OA), Fontana-Masson silver stain, Leishman stain, Periodic Acid-Schiff (PAS) and Giemsa have been used for the diagnosis of *T. vaginalis.*^17,48,50,51^ However, these techniques are associated with a loss of motility and morphologic characteristics during the fixation and staining steps.^17,49,50^ Furthermore, it is difficult to interpret the stained smears because *T. vaginalis* can resemble polymorphonuclear leucocytes.^17,49^ Papanicolaou (Pap) smear has a sensitivity of ~60%^13,52^ but a specificity of 95%.^52^ Moreover, this technique is associated with false-positive and false-negative results.^17,50^

#### Culture

For the past decades, the broth culture technique has been considered as the gold standard for the detection of *T. vaginalis* infection.^17,49,50,53^ Culture media such as Kupferberg Trichosel medium, Kupferberg STS medium, Difco Kupferberg medium, Lash serum medium and Diamond medium have been used to grow *T. vaginalis in vitro*.^54^ The standard broth is Diamond’s medium,^50^ and it only requires an inoculum size of 300 organisms/mL – 500 organisms/mL.^17,48,51^

The advantages of broth culture technique include detection of relatively few organisms,^49^ and it is easy to interpret.^17,49,50^ However, this technique is expensive,^10,13,50^ insensitive in men and time consuming.^10^ Broth culture normally requires 2–7 days of incubation^13,17,50^ and the culture needs to be examined by wet mount microscopy every day.^48^ Furthermore, bacterial contamination is common in cultures, which interferes with the detection of *T. vaginalis.*^50^

The InPouch™ system (BioMed Diagnostics, United States) is an improvement of the broth culture and wet mount techniques. The InPouch™ system allows for an easy inoculation, immediate observation, storage and transport of the specimen as well as microscopic observation inside the device.^50,51,55^ The InPouch^™^ medium contains salts, maltose and other sugars, peptones, amino acids and antibiotics in a phosphate buffered saline (PBS) base. The InPouch™ device is an oxygen-resistant plastic pouch, which has two chambers joined together by a thin passage. The top chamber acts as the slide used for wet mount microscopy.^51,55,56^ If microscopic examination is negative, the fluid in the top chamber is squeezed into the bottom chamber and further incubated.^51,56^ This system has a sensitivity between 81% and 94% and a specificity of 100%.^55^

The cell culture technique uses different cell lines to detect *T. vaginalis* from clinical specimen.^17,51^ Cell culture technique requires the specimen to be treated with antimicrobials prior to passage onto the cell culture.^50^ Cell culture has high sensitivity and it only requires an inoculum of three trichomonads/mL.^17,50^ However, this technique is more prone to bacterial contamination,^50^ expensive and not suitable for rapid and routine diagnosis of *T. vaginalis.*^17,50^

#### Molecular techniques

Several recombinant deoxyribonucleic acid (DNA) technology assays have been developed for the diagnosis of trichomoniasis.^50^ The Affirm VP III (Becton, Dickinson & Co, United States) has been approved for *T. vaginalis* diagnosis in women by the United States Food and Drug Administration (USFDA or simply FDA).^10,53,57^ The Affirm VP III test is used for the detection of *Gardnerella vaginalis, T. vaginalis*^10^ and *Candida albicans*, and the results are available in approximately 45 min.^10,53,57^ However, this test might be associated with false positives because of the presence of DNA from dead *T. vaginalis* after treatment.^50^ Affirm VP III has a sensitivity of ~83% and a specificity of ~97%.^10,53,58^ The APTIMA Analyte Specific Reagents test (ASR, manufactured by Gen-Probe, Inc.) was approved by the USFDA in 2011 for the detection of *T. vaginalis.*^58^ Studies have reported a sensitivity of 74% – 98% and a specificity of 87% – 98%.^10,53,58^ Amplicor (Roche Diagnostic Corp.) used for *C. trachomatis* and *N. gonorrhoea* infections has been modified for *T. vaginalis* diagnosis. This polymerse chain reaction (PCR) assay can detect *T. vaginalis* in urine, vaginal and endocervical specimen of both men and women.^10,53^ The Cepheid GeneXpert system (Sunnyvale, CA, United States) is already approved for use to diagnose STIs, such as *C. trachomatis* and *N. gonorrhoeae.*^59^ Schwebke et al. (2018) recently showed that the Cepheid Xpert *T. vaginalis* assay has a sensitivity of 97.2% and a specificity of 99.9% compared with the InPouch culture assay.^59^ The Xpert TV assay can be used on urine specimen for both men and women, endocervical swabs and self-collected vaginal swabs in asymptomatic and symptomatic patients. This system is easy to use and the results are available in 63 min with early termination within 40 min for positive results, and therefore it can be used at primary health care settings as a point-of-care (POC) test.^59^

#### Immunological techniques

Trichomonal antibodies can be detected by using several techniques including enzyme-linked-immunosorbent serologic assay, complement fixation, haemagglutination, immune fluorescence test (IFT) and gel diffusion. However, these techniques cannot distinguish recent and past infections.^17,49,50^ Trichomonas Direct Enzyme Immunoassay (California Integrated Diagnostics, Benicia) and Fluorescent Direct Immunoassay detect trichomonal antigens. These assays use fluorochrome- and peroxidase-labelled monoclonal antibodies to detect the antigens of *T. vaginalis.*^17^ Results of these assays are available in 1 h, and thus the diagnosis and treatment of the patient are possible in a single visit.^17^ The OSOM test is based on immune-chromatographic capillary flow dipstick technology,^10,51,53,58,60^ and the results are available in approximately 10 min.^10,53,58^ This POC test has a sensitivity of approximately 83% and a specificity of approximately 97%.^10,51,53,58^ The major drawback of this test is that it cannot be used for men.^60^

### Impact on women’s health

#### Global

Numerous studies have also shown a positive association between *T. vaginalis* infection and HIV acquisition. *Trichomonas vaginalis* increases the risk of acquiring HIV by an estimated two-fold.^61^ The biological reasons for *T. vaginalis* increasing HIV acquisition amongst women could be because of two reasons: (1) the accumulation of both macrophages and cluster of differentiation 4 (CD4) lymphocytes, which are HIV target cells and (2) the disruption of the vaginal epithelial barrier enabling the movement of HIV into the laminae propriae.^62^ The high prevalence of *T. vaginalis* amongst women may be associated with a higher incidence of HIV globally.^58^ *Trichomonas vaginalis* infection is also associated with increased HIV transmission.^63^ Because *T. vaginalis* infection is asymptomatic in the majority of cases and often remains untreated, this infection can be easily transmitted.^61^

Trichomoniasis has been associated with health complications such as adverse pregnancy outcomes, PID, neoplasia^58^ and co-infection with other infections such as HIV, bacterial vaginosis (BV) and high-risk (HR) human papillomavirus (HPV)-16 genotype.^64^ Adverse pregnancy outcomes include preterm delivery, low birth weight,^15,16,65^ neonatal morbidity and mortality.^65^ *Trichomonas vaginalis* infection can be acquired in new-born infants during birth.^2,65^ It has been reported that approximately 25 million pregnant women have trichomoniasis^66^ and 2% – 17% of female infants acquire *T. vaginalis* infection through direct vulvo-vaginal infection.^65^

#### Africa

*Trichomonas vaginalis* has been associated with increased genital shedding of HIV.^63^ A past study found a high prevalence of *T. vaginalis* amongst HIV sero-discordant African couples.^67^ A population-based survey undertaken in rural and peri-urban KwaZulu-Natal, South Africa, reported a co-infection rate of 18.1% for *T. vaginalis* and HIV in women.^68^ A high prevalence of *T. vaginalis* (20%) in a population of HIV-infected pregnant women seeking antenatal care at public health centres in South Africa was reported.^69^ In a cohort of South African women attending primary health care facilities, *T. vaginalis* was shown to be significantly associated with an HIV-positive status. According to that study, *T. vaginalis* infection was present in almost 25% of HIV-infected women.^6^ In women with trichomoniasis, there is a risk of co-infection with BV.^70^ A study conducted by Abbai et al. (2016) in South African women showed a significant association between baseline BV infections and incident *T. vaginalis* infections.^71^

Cervical cancer is the most common cause of cancer-related deaths in young women from sub-Saharan Africa.^72^ Genital HPV infection, one of the common viral STIs, has been linked to cervical cancer in women.^73^ Genital HPV genotypes are classified into either ‘HR’ or ‘low-risk’ (LR).^74^

It is suggested that *T. vaginalis* infection might be associated with an increased risk of cervical cancer, and a history of *T. vaginalis* infection has been shown to be a risk factor for HPV infection.^75^ In a population of South African women, *T. vaginalis* was shown to be prevalent in women diagnosed with cervical intra-epithelial neoplasia (CIN).^76^ A study conducted by Lazenby et al. (2014) in a population of Tanzanian women reported that *T. vaginalis* was associated with an increased risk of HR HPV.^72^ Women with *T. vaginalis* were 6.5 times more likely to have HPV type 16 when compared with women without *T. vaginalis.*^72^

Pelvic inflammatory disease is the inflammation of the upper genital tract structures caused by ascension of microbes from the lower genital tract.^77^ Approximately half of PID cases are attributable to gonorrhoea and chlamydia, whilst the remainder are of unknown aetiology.^78^ A study conducted amongst South African women found a strong association between PID and *T. vaginalis* infection.^32^ In that study, women with trichomoniasis had a significantly higher risk of PID when compared with women without infection. In addition, this association was exacerbated in the presence of HIV infection.^32^

Despite the estimated large burden of *T. vaginalis* infection in the African region, data on clinical outcomes associated with this infection are limited. However, in other regions of the world, multiple studies have been conducted on the association between *T. vaginalis* infections and morbidity. More studies conducted in African cohorts are urgently needed to provide data on the morbidity associated with *T. vaginalis* infection.

### Treatment and emerging challenges

Currently, the 5-nitroimidazoles are the only drugs used for treatment of trichomoniasis.^79^ Metronidazole and tinidazole are the only drugs cleared by the FDA for the trichomoniasis therapy.^20,62,79^ Metronidazole has been the standard and approved treatment for trichomoniasis since the 1960s^17,79,80,81^ and in some countries, it is the only drug that is approved for the treatment of trichomoniasis.^82^ Nitroimidazoles are heterocyclic compounds that possess a nitro group on the fifth position of an imidazole ring. Metronidazole is a small molecule, and it enters the trophozoite cells through passive diffusion.^12,51,81^ This drug is inactive, and it is reduced anaerobically in the hydrogenosome of the trichomonad by pyruvate-ferredoxin oxidoreductase (PFOR).^17,51,81,83^ Pyruvate-ferredoxin oxidoreductase is involved in the oxidative decarboxylation of pyruvate to form acetyl–coenzyme A, a crucial step in many metabolic pathways, in most anaerobes.^84^

Metronidazole acts as an electron acceptor by accepting electrons from the reduced ferredoxin, resulting in the formation of cytotoxic nitro radicals.^51^ The nitro radicals bind to DNA, which leads to the breaking of strands, subsequently leading to a cell death of the parasite.^17,51,80,81^ In *T. vaginalis*, flavin reductase is part of its antioxidative defence. Flavin reductase indirectly reduces molecular oxygen to hydrogen peroxide via free flavins. A reduced or absent flavin reductase activity has been reported in metronidazole-resistant *T. vaginalis.*^85^

The current WHO and Centers for Disease Control and Prevention (CDC) guidelines for the treatment of trichomoniasis include: a metronidazole or tinidazole single dose of 2 g and an alternative dose of metronidazole 400 mg – 500 mg twice a day for 7 days.^20,58,83^ Metronidazole is classified as an FDA pregnancy category B drug and treatment with 2 g at any stage of pregnancy is recommended.^10,58,62^ Tinidazole has not been evaluated for use during pregnancy; therefore, it is classified as category C.^58,62^ The recommended metronidazole and tinidazole regimens have cure rates of approximately 95%^20,58,86^ and 86% – 100%,^20,51,86^ respectively. Metronidazole (500 mg) is also available as intravaginal ovules (Flagyl) or tablets (Tergynan), which is used once a day for 10 days.^79^ Miltefosine and nitazoxanide have been shown to be effective against *T. vaginalis in vitro*. However, the safety of miltefosine during pregnancy is yet to be reported and nitazoxanide may only be used as an intravaginal treatment option as it is poorly absorbed in the intestinal tract.^87^ Reports on metronidazole resistance of *T. vaginalis* strains have been increasing.^1^ Even though the metronidazole cure rates are high, clinical treatment failure is challenging.^17^ Most *T. vaginalis* isolates are highly susceptible to metronidazole; however, laboratory resistance and treatment failure have been reported.^18,19^ In South Africa, metronidazole resistance was reported in 6% (2/30) of the *T. vaginalis* strains isolated from women attending the antiretroviral clinic at the Tshwane District Hospital, Pretoria.^24^ A more recent study conducted in South African antenatal women reported that 9.5% (2/21) of the *T. vaginalis* isolates obtained were resistant to metronidazole.^21^

Clinical metronidazole resistance is defined as failure to cure the infection after two consecutive courses of treatment.^51,86^ Treatment failure may be because of insufficient absorption and/or delivery of metronidazole to the target site, inactivation of the drug by vaginal flora, trichomonad-dependent activity and reinfection or non-compliance.^17,20,86^

### Prevention efforts for *Trichomonas vaginalis* infections

#### Condom use

The practice of condom use is an important component of STI control programmes.^88^ Condoms prevent infection acquisition and transmission. Consistent condom use has been shown to reduce *T. vaginalis* infections in women.^89^ Reports from several surveys conducted in African countries showed that women used condoms less consistently than men.^90^ This inconsistent condom use was largely because of religious beliefs and the inability of women to negotiate condom use with their male partners.^91^ A study conducted in adolescent and young women from Kenya reported that having receptive vaginal sex without a condom was significantly associated with the *T. vaginalis* infection.^92^ In South Africa, a high burden of STIs including *T. vaginalis* was reported in women who lacked condom use.^93^

#### Partner notification

An important component of STI management includes partner notification, which will possibly reduce re-infection and prevent STI-related health complications.^1^ A study conducted on pregnant women in Cape Town, South Africa, observed that younger maternal age was associated with partner notification and treatment; however, partner treatment was low.^94^ Similarly, a study conducted in Gaborone, Botswana, on pregnant women found that pregnant women are willing to utilise patient-based partner notification; however, partner treatment is low.^95^ A study conducted on youth aged 16–24 years in Durban, South Africa, who were assessed for STI diagnosis, treatment and partner notification, reported that stigma and lack of STI knowledge were reasons for not notifying their partner of STIs.^96^ A systematic review of studies conducted in sub-Saharan Africa on the diagnosis and treatment of any curable STIs with partner notification being the outcome found that direct patient referral is the most commonly used and evaluated partner notification strategy for STIs. However, there is discordancy amongst the studies reviewed, which calls for future research that will investigate other methods such as expedited partner treatment.^97^

#### Vaccines

Risky sexual behaviour such as lack of condom use and having multiple sexual partners negate efforts in controlling the global burden of trichomoniasis.^46^ Vaccination against *T. vaginalis* would provide a solution for control efforts.^79^ The priority to develop a vaccine against *T. vaginalis* has been low when compared with other human parasitic protozoans.^98^ Founding research on vaccine development for *T. vaginalis* began in the 1960s; 100 women affected with resistant trichomoniasis were treated by using heat-killed *T. vaginalis* administered by intravaginal inoculation.^99^ The study by Aburel et al. (1963) reported a 40% elimination of *T. vaginalis* in the infected women.^99^ Unfortunately, a similar trial has never been conducted to find an effective vaccine against this pathogen.^98^ Data from two pre-clinical trials using animal models in which mice were immunised with whole *T. vaginalis* cells emulsified in adjuvant showed some protection against the pathogen.^100,101^ A study conducted by Hernández et al. (2005) showed that intranasal immunisation with a 62-kDa proteinase purified from *T. vaginalis* resulted in improved eradication of *T. vaginalis* following intravaginal challenge of BALB/c mice.^102^ Newer research into the development of *T. vaginalis* vaccines have focussed on the screening of potential immunogens.^98^

#### Vaginal products

The vaginal route may be an appropriate site for drug delivery. Administration of drugs through the vagina may result in better bioavailability after administration when compared with oral administration.^103^ There are various pharmaceutical products that can be administered through the vaginal administration and these include liquid solutions, emulsions, suspensions and solids, such as pessaries, vaginal tablets, vaginal capsules and vaginal films.^79^ A metronidazole vaginal gel has also been tested; however, studies have shown that the gel is effective in only approximately half of the reported trichomoniasis cases.^81^ A study was conducted, which compared the treatment for trichomoniasis with vaginal tablets with a low dosage of metronidazole (100 mg) and oral metronidazole (500 mg twice a day) for 7 days. The study showed that treatment with vaginal tablets containing 100 mg of metronidazole resulted in a 64% cure rate.^104,105^

## Conclusion

Based on the high global prevalence of *T. vaginalis* and associated health consequences, there is a need for improved screening programmes that will lead to early diagnosis, detection of asymptomatic infections and effective treatment regimens. In addition, metronidazole treatment failure should also be detected early and managed appropriately. The emerging patterns of drug resistance that have been observed call for newer efforts regarding vaccine design and the development of different drug formulations.

### Recommendations

The syndromic case management remains the foundation for STI treatment in Africa. However, there are several disadvantages of syndromic management such as failing to treat asymptomatic infections, over-treatment (resulting in antibiotic resistance), as well as poor sensitivity and specificity of algorithms in accurately diagnosing the infections, specifically for women.^106^ The syndromic management algorithms for vaginal discharge have a low positive predictive value for STI pathogens. Screening and surveillance programmes for STIs are urgently needed to assess the true burden of these infections in the African region. Given that a high proportion of STIs are asymptomatic, inclusion of STI screening services within HIV-testing facilities may lead to early diagnosis, treatment and prevention of both HIV and STIs in at-risk populations. To obtain accurate prevalence estimates for infection (asymptomatic and symptomatic), diagnostic testing will need to be implemented. However, to implement a test and treatment programme in resource-limited settings, appropriate, affordable, accurate and rapid POC tests are needed.
